# Supplementary material for: A computationally-enhanced hiCLIP atlas reveals Staufen1-RNA binding features and links 3′ UTR structure to RNA metabolism
Source: Nucleic Acids Res. 2023 Apr 4;51(8):3573–89. doi: 10.1093/nar/gkad221 (PMC10164587; doi:10.1093/nar/gkad221)
Supplement: gkad221_Supplemental_File [file gkad221_supplemental_file.pdf]

SUPPLEMENTARY FIGURE LEGENDS

Suppl. Fig. S1: A workflow giving an overview of the main steps of the *Tosca* computational pipeline.

Suppl. Fig. S2: Analyses of *in silico* proximity ligation reads constructed from linker-containing hybrid reads.

(A) Above: proportion of *in silico* reads recovered as hybrids using the Tosca pipeline. Below: the breakdown of recovered hybrids by whether they match the known true hybrid solutions.

(B) Read length distributions for recovered and not recovered hybrids across RNase conditions.

(C) Lengths of the shorter arms of hybrid reads for recovered and not recovered hybrids across RNase conditions.

(D) Proportions of inter-transcript and intra-transcript hybrids for recovered and not recovered hybrids

(E) Regional distribution of arms for recovered and not recovered hybrids

Suppl. Fig. S3:

(A) Silhouette method applied to the k-means clustering for Fig. 3C to identify the optimal number of clusters

(B) Mean pairing probability profiles for the 3 clusters identified in Fig. 3C. Dashed blue lines indicated local minima used to delineate the proximal and distal arm sequences.

Suppl. Fig. S4:

IGV screenshot of STAU1 hybrids on the ARF1 transcript, visualised using the atlas BAM file and grouped by cluster using the “CL” tag and coloured by read orientation using the “RO” tag.

Suppl. Fig. S5:

(A) Overlap between STAU1 hiCLIP duplexes and PARIS 3’ UTR intra-transcript proximity ligation duplexes (left) and RIC-seq 3’ UTR intra-transcript interactions (right).

(B) Two-component Gaussian mixture model that identifies two duplex span size distributions for the STAU1 hiCLIP proximity ligation duplexes: red: $\mu = 18$, $\lambda = 0.46$; blue: $\mu = 252$, $\lambda = 0.53$.

(C) Hybrid arm length for all hybrid reads from hiCLIP, PARIS and RIC-seq

(D) Number of bulges per duplex for 3’UTR intra-transcript duplexes.

(E) Percentage of bulges with a symmetrically positioned and identical size bulge in the other arm within the duplexes.

(F) Longest bulge-free stem segment within the duplexes.

(G) Arc plot of the STAU1 hiCLIP and PARIS duplexes on the *SRSF1* 3’ UTR, alongside STAU1 crosslinking signal and transcriptomic annotation; the duplexes used in Fig. 5I as representative for PARIS and STAU1 are indicated with a dagger and double dagger respectively.

Suppl. Fig. S6:

(A) RNA expression for genes with STAU1 3’ UTR duplexes, global 3’ UTR duplexes identified by PARIS (but not STAU1 hiCLIP) or without any identified 3’ UTR duplexes.

(B) RNA metabolism (synthesis, processing and degradation rates) as measured by (5) for the genes as in (A), with those without any identified 3’ UTR duplexes subset to match the RNA expression of those with identified 3’ UTR duplexes either by STAU1 hiCLIP or PARIS.

(C) Correlation between RNA degradation calculations in (5) and RNA half-life (HL) measurements in (26) from experiments after transcriptional shut-off with actinomycin D (ActD) and α-amanitin (Aman) and after 4sU labelling and calculated with DRUID (26). Spearman’s correlations for each comparison are enumerated in the top right of each panel.

(D) RNA degradation rates reproduced clustered as in Figure 6B, alongside which are shown the centred and scaled half-life calculations from (26).

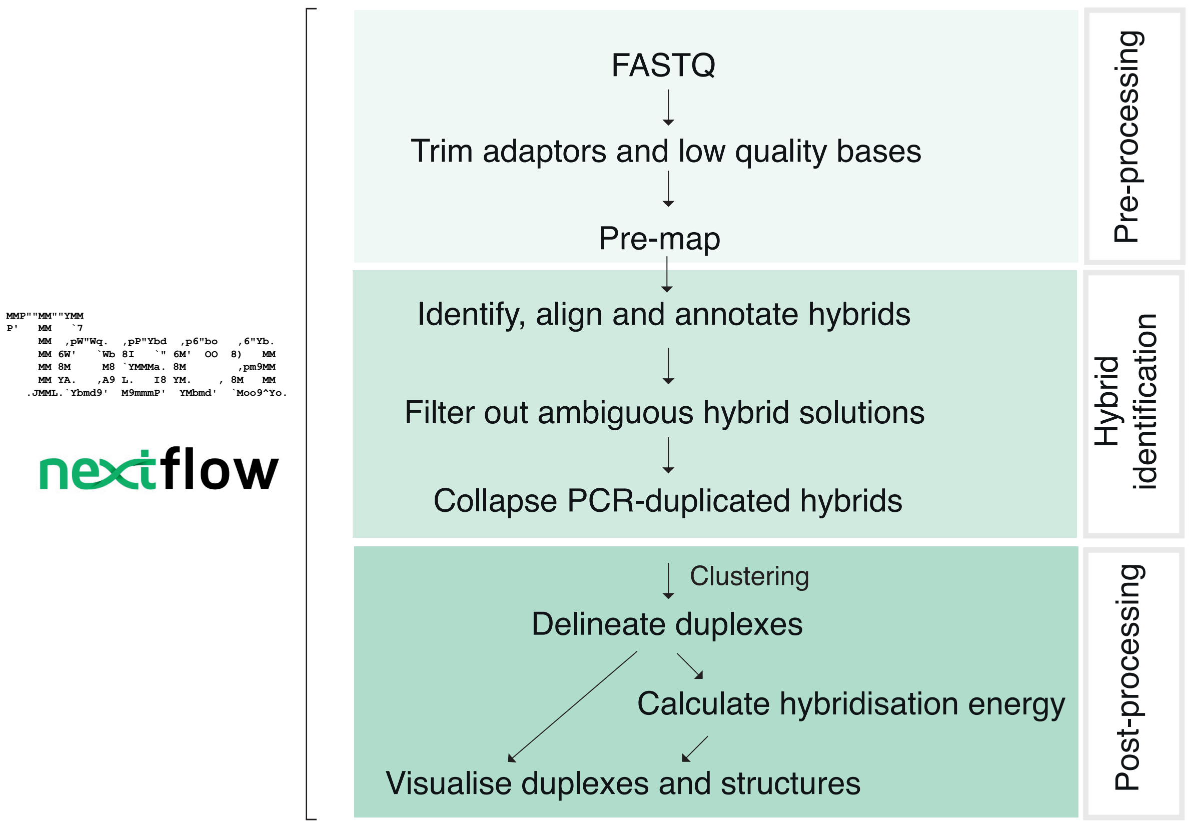
nextflow
FASTQ
Trim adaptors and low quality bases
Pre-map
Pre-processing
Identify, align and annotate hybrids
Filter out ambiguous hybrid solutions
Collapse PCR-duplicated hybrids
Hybrid identification
Clustering
Delineate duplexes
Calculate hybridisation energy
Visualise duplexes and structures
Post-processing

Suppl. Fig. S1

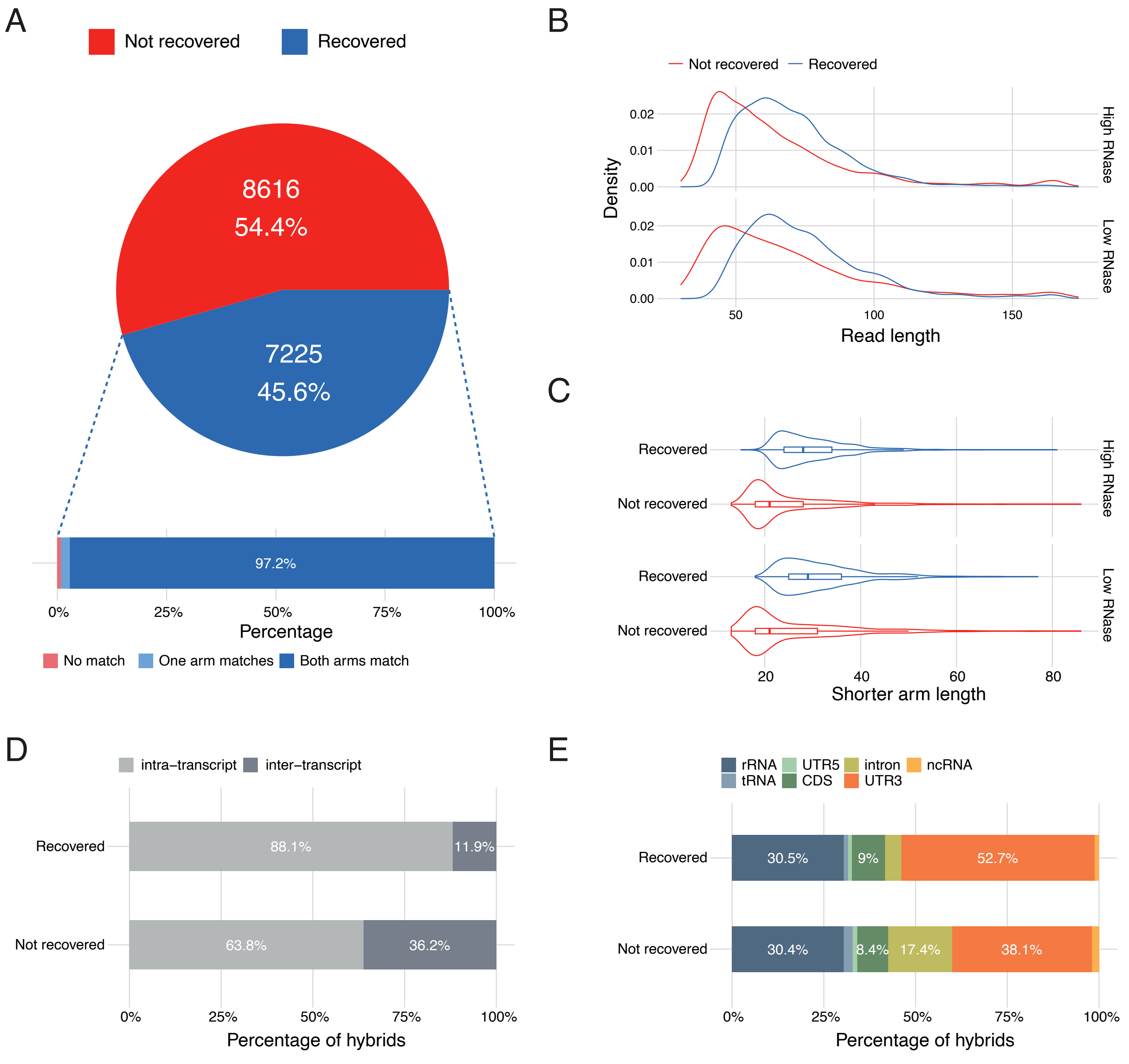
A
Not recovered
Recovered
8616
54.4%
7225
45.6%
97.2%
0%
25%
50%
75%
100%
Percentage
No match
One arm matches
Both arms match
B
Not recovered
Recovered
Density
0.02
0.01
0.00
High RNase
Low RNase
50
100
150
Read length
C
Recovered
Not recovered
High RNase
Low RNase
20
40
60
80
Shorter arm length
D
intra−transcript
inter−transcript
Recovered
Not recovered
88.1%
11.9%
63.8%
36.2%
Percentage of hybrids
E
rRNA
tRNA
UTR5
CDS
intron
UTR3
ncRNA
30.5%
9%
52.7%
30.4%
8.4%
17.4%
38.1%
Percentage of hybrids

Suppl. Fig. S2

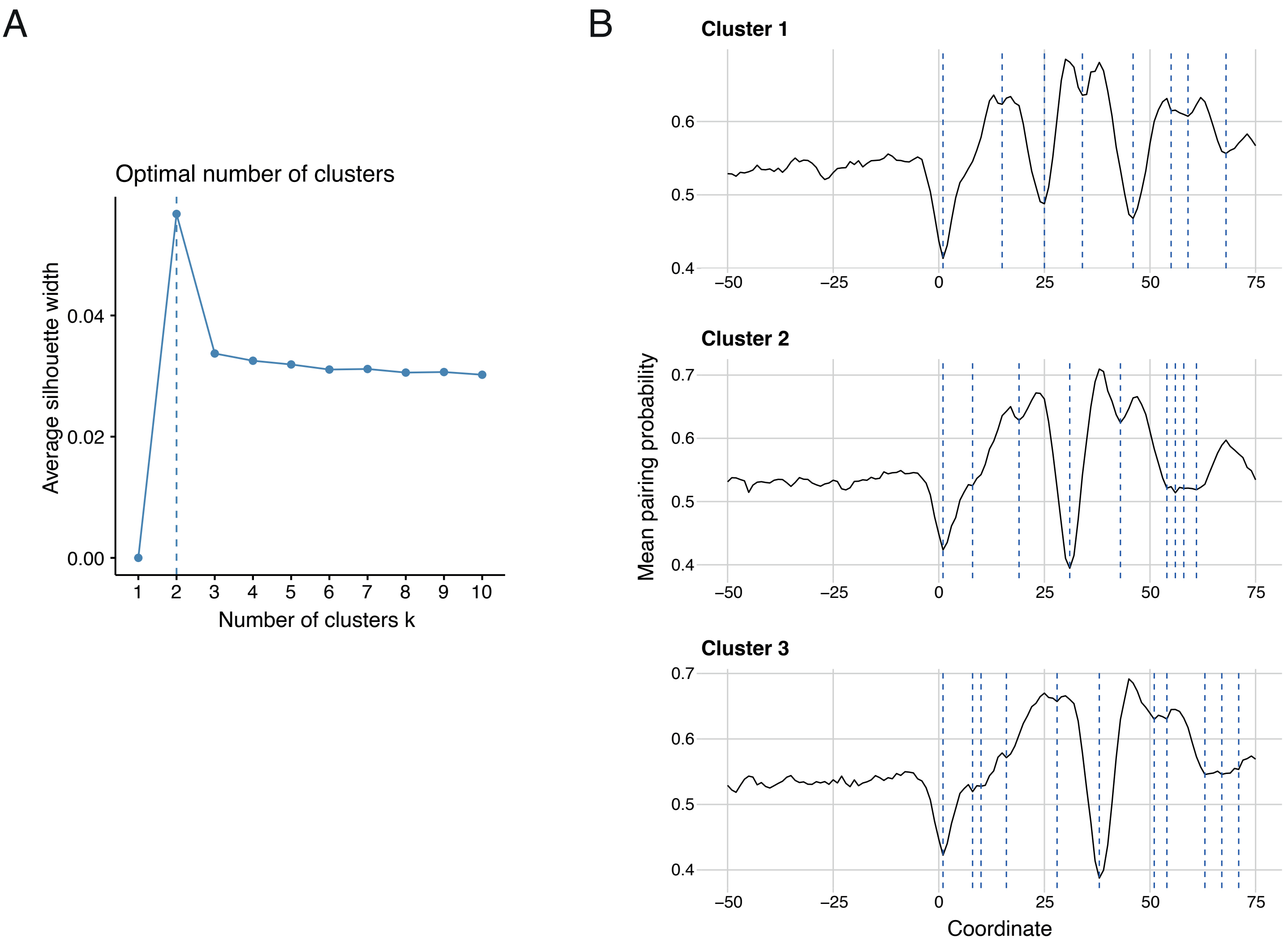
A
Optimal number of clusters
Average silhouette width
0.00
0.02
0.04
1
2
3
4
5
6
7
8
9
10
Number of clusters k
B
Cluster 1
Cluster 2
Cluster 3
Mean pairing probability
0.4
0.5
0.6
0.7
−50
−25
0
25
50
75
Coordinate

Suppl. Fig. S3

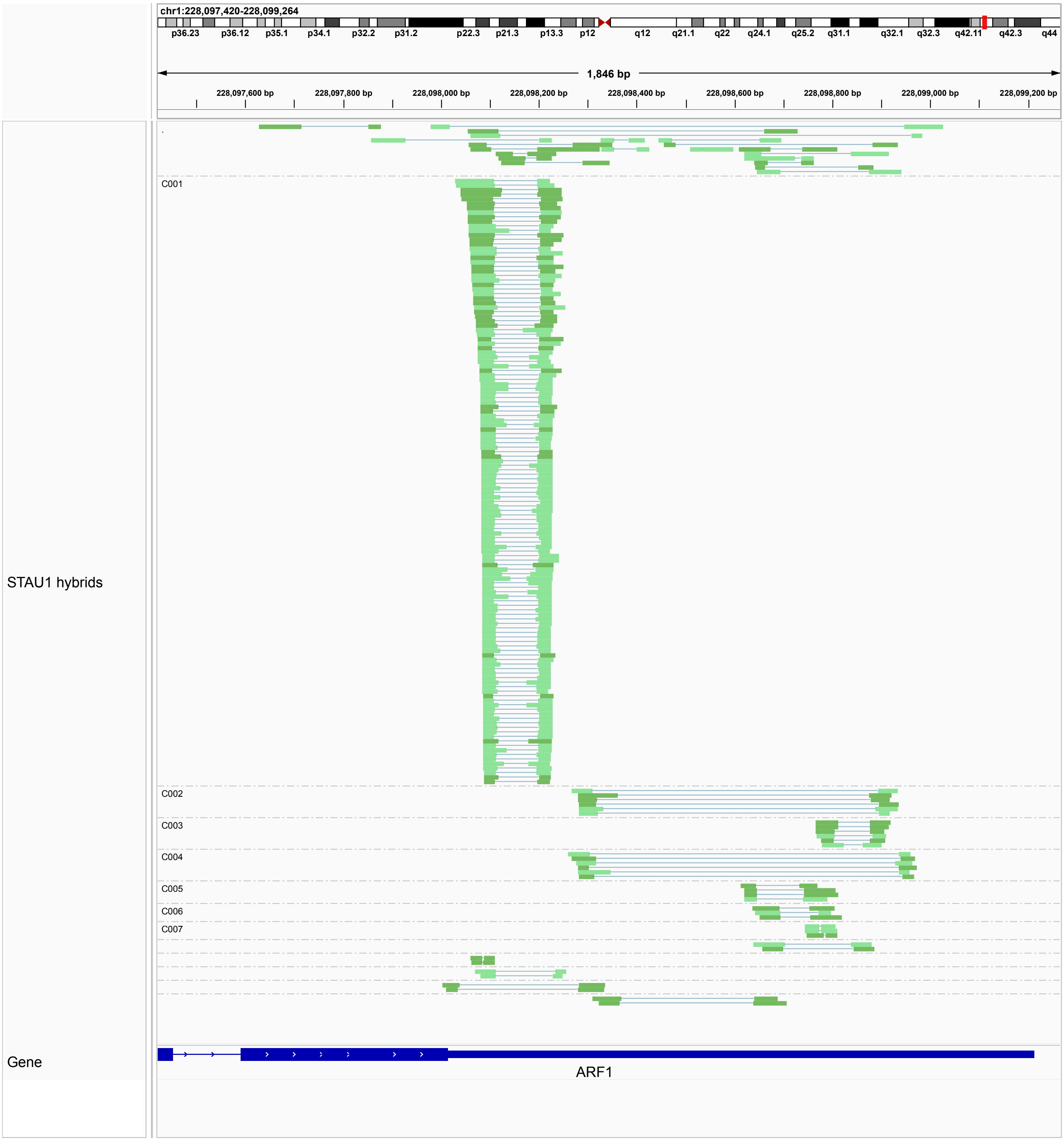
chr1:228,097,420-228,099,264
p36.23 p36.12 p35.1 p34.1 p32.2 p31.2 p22.3 p21.3 p13.3 p12 q12 q21.1 q22 q24.1 q25.2 q31.1 q32.1 q32.3 q42.11 q42.3 q44
1,846 bp
228,097,600 bp 228,097,800 bp 228,098,000 bp 228,098,200 bp 228,098,400 bp 228,098,600 bp 228,098,800 bp 228,099,000 bp 228,099,200 bp
STAU1 hybrids
C001
C002
C003
C004
C005
C006
C007
Gene
ARF1

Suppl. Fig. S4

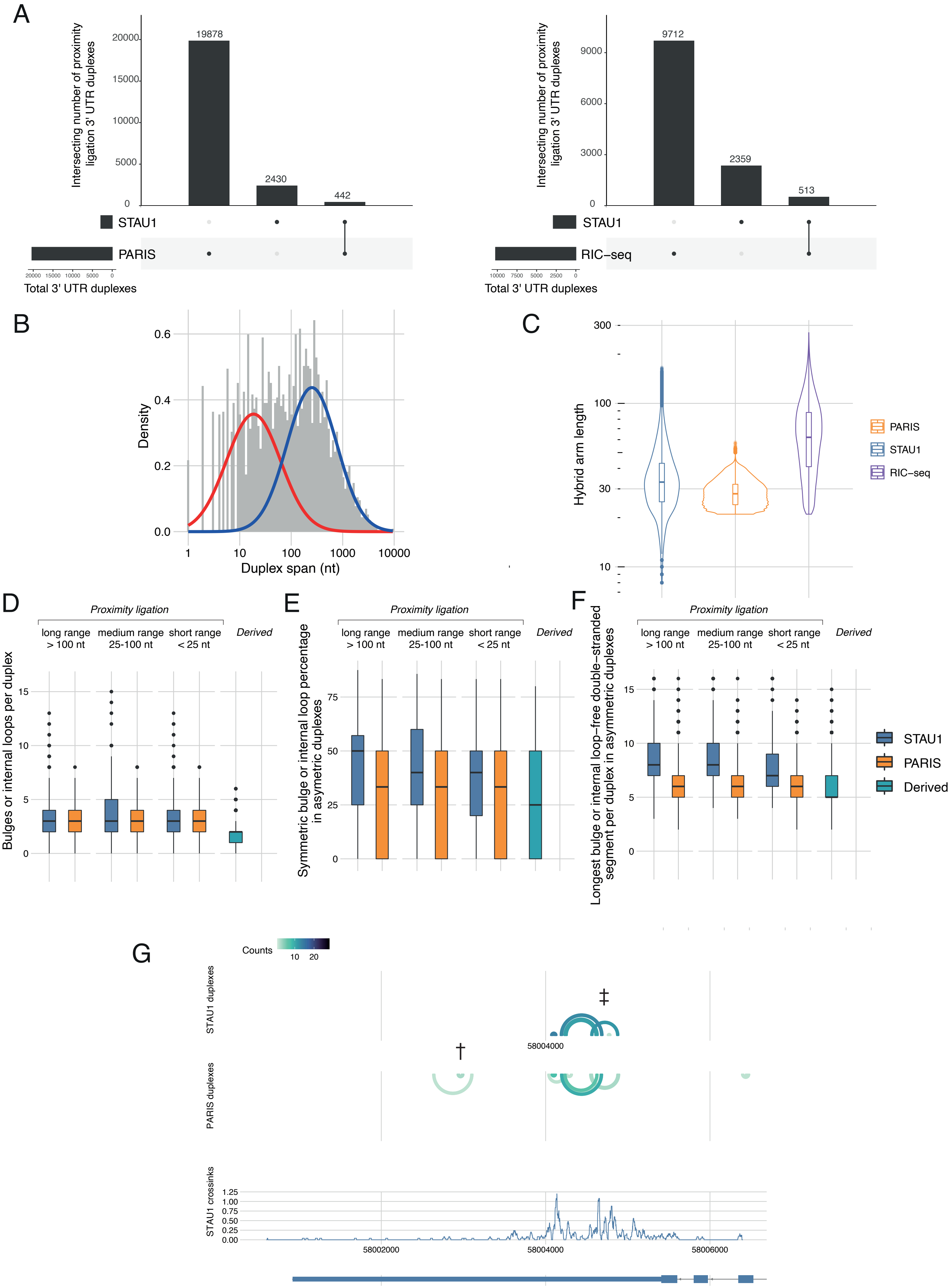
A
Intersecting number of proximity ligation 3' UTR duplexes
19878
2430
442
STAU1
PARIS
Total 3' UTR duplexes
9712
2359
513
STAU1
RIC−seq
Total 3' UTR duplexes
B
Density
Duplex span (nt)
C
Hybrid arm length
PARIS
STAU1
RIC−seq
D
Proximity ligation
long range > 100 nt
medium range 25-100 nt
short range < 25 nt
Derived
Bulges or internal loops per duplex
E
Proximity ligation
long range > 100 nt
medium range 25-100 nt
short range < 25 nt
Derived
Symmetric bulge or internal loop percentage in asymetric duplexes
F
Proximity ligation
long range > 100 nt
medium range 25-100 nt
short range < 25 nt
Derived
Longest bulge or internal loop−free double−stranded segment per duplex in asymmetric duplexes
STAU1
PARIS
Derived
G
Counts
STAU1 duplexes
‡
†
58004000
PARIS duplexes
STAU1 crossinks
58002000
58004000
58006000

Suppl. Fig. S5

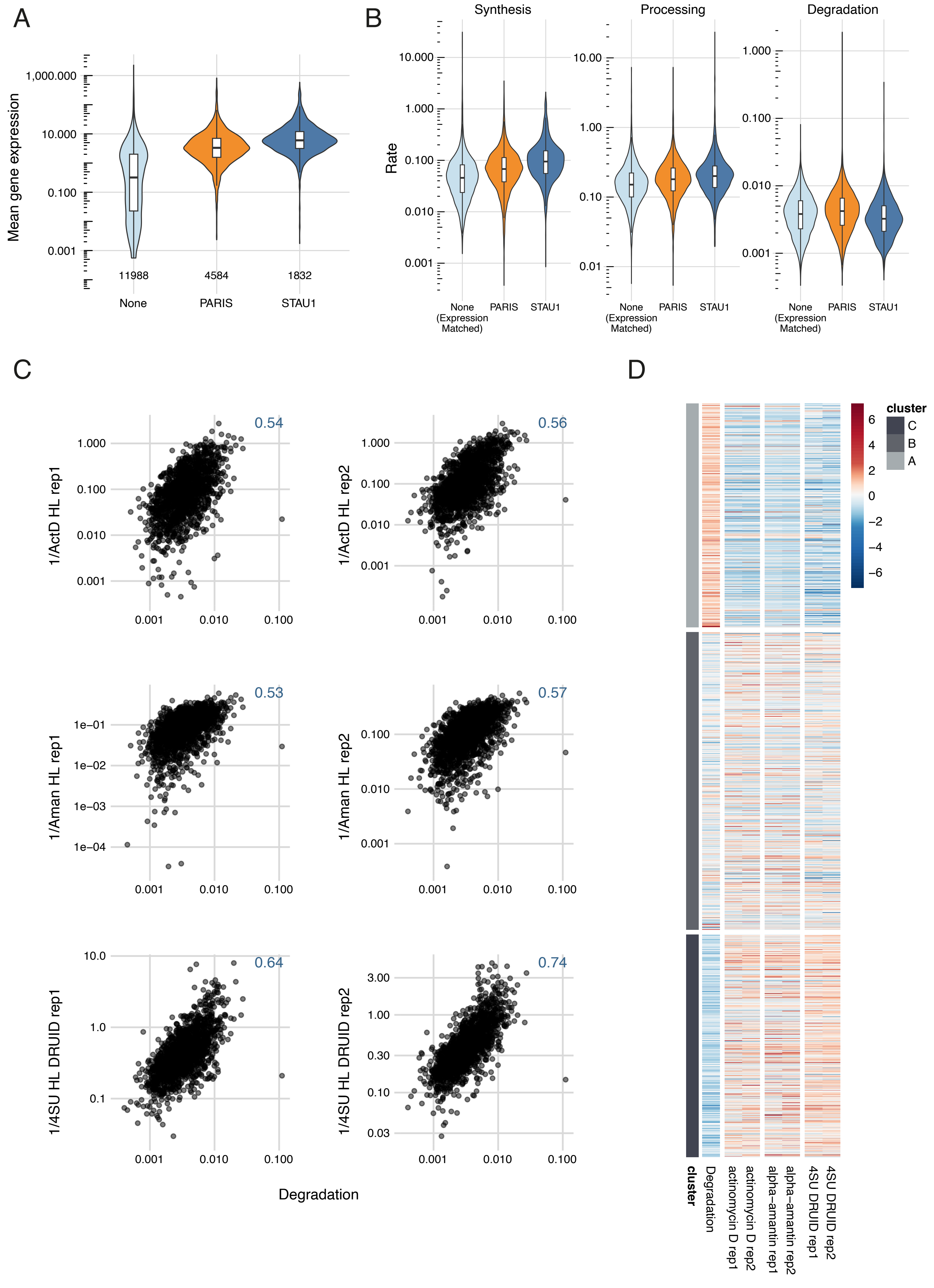
A
B
Synthesis
Processing
Degradation
Mean gene expression
Rate
11988
4584
1832
None
PARIS
STAU1
None (Expression Matched)
C
D
0.54
0.56
0.53
0.57
0.64
0.74
1/ActD HL rep1
1/ActD HL rep2
1/Aman HL rep1
1/Aman HL rep2
1/4SU HL DRUID rep1
1/4SU HL DRUID rep2
Degradation
cluster
C
B
A
actinomycin D rep1
actinomycin D rep2
alpha−amantin rep1
alpha−amantin rep2
4SU DRUID rep1
4SU DRUID rep2

Suppl. Fig. S6
